# Short-term effect of fenofibrate on C-reactive protein: A meta-analysis of randomized controlled trials

**DOI:** 10.1186/1758-5996-3-24

**Published:** 2011-09-22

**Authors:** Jiatao Ye, James N Kiage, Donna K Arnett, Alfred A Bartolucci, Edmond K Kabagambe

**Affiliations:** 1 Department of Epidemiology, University of Alabama at Birmingham, Birmingham, AL, USA; 2 Department of Biostatistics, University of Alabama at Birmingham, Birmingham, AL, USA

**Keywords:** CRP, fenofibrate, meta-analysis, randomized, clinical trials, short-term

## Abstract

**Background:**

C-reactive protein (CRP) is positively associated with risk for cardiovascular disease and all-cause mortality. Some but not all randomized and non-randomized clinical trials found significant associations between fenofibrate therapy and CRP but the direction and magnitude of the association varied across studies. The duration of treatment, patient populations and sample sizes varied greatly, and most short-term studies (i.e., ≤ 12 weeks) had fewer than 50 patients. In this study we meta-analyzed randomized clinical trials to determine the short-term effect of fenofibrate on CRP.

**Methods:**

Two reviewers independently searched PubMed and other online databases for short-term randomized clinical trials that reported CRP concentrations before and after fenofibrate treatment. Of the 81 studies examined, 14 studies with 540 patients were found eligible. Data for the change in CRP and corresponding measures of dispersion were extracted for use in the meta-analysis.

**Results:**

The weighted mean CRP concentrations before and after fenofibrate therapy were 2.15 mg/L and 1.53 mg/L (-28.8% change), respectively. Inverse-variance weighted random effects meta-analysis revealed that short-term fenofibrate treatment significantly lowers CRP by 0.58 mg/L (95% CI: 0.36-0.80). There was significant heterogeneity between studies (Q statistic = 64.5, *P*< 0.0001, I^2 ^= 79.8%). There was no evidence of publication bias and sensitivity analysis revealed that omitting any of the 14 studies did not lead to a different conclusion from the overall meta-analysis result.

**Conclusion:**

Short-term treatment with fenofibrate significantly lowers CRP concentration. Randomized trials that will recruit patients based with high baseline CRP concentrations and with change in CRP as a primary outcome are needed.

## 1. Introduction

C-reactive protein (CRP), a nonspecific marker of inflammation, is a predictor of incident cardiovascular events and mortality [1-5]. Although the exact mechanism through which CRP leads to increased risk for adverse cardiovascular events is unknown, many studies indicate that reducing serum CRP concentration leads to significant benefits with regard to the future cardiovascular events [6,7]. Whether the reduction in events is directly due to the reduction in CRP or due to the concomitant improvement in lipid profiles remains to be elucidated.

In addition to genetic determinants [4,8], other factors such as age, race, gender, BMI, smoking, alcohol use, diet and exposure to pollutants are known to affect CRP concentrations in serum [4,9]. Other than genetic factors, age, race and gender, all the other risk factors for elevated serum CRP are potentially modifiable and they provide an opportunity for intervention. Indeed, studies have shown that exercise, dietary modifications as well as other lifestyle changes lead to a reduction in serum CRP levels [10,11]. Despite this apparent benefit, exercise, dietary and lifestyle changes have only proven to be effective on a short-term basis because of poor compliance as people tend to revert to their old habits [10]. Lifestyle changes require highly motivated individuals in order to attain long-term benefits, an attribute that limits their application in management of chronic inflammation.

Pharmacologic interventions may provide an effective strategy to manage elevated CRP concentrations, especially among individuals who are not willing or are unable to adhere to dietary and other lifestyle changes [4]. Statins such rosuvastatin or simvastatin are effective in lowering serum CRP concentrations [4,9,12-14]. Other studies have shown that niacin, fish oil esters and fenofibrate individually or in combination have an effect on serum CRP concentrations [15]. However, the results from fenofibrate trials have been inconsistent, with some studies indicating a reduction [13,15] while others indicating no change or an increase in CRP [16-18]. A number of factors may explain this apparent inconsistency in outcome. First, most of the studies may have been underpowered (typically < 50 patients per study). Second, the dose and formulation of fenofibrate varied greatly. Although the different formulations could have been metabolically equivalent regardless of the dose, the associations between fenofibrate and CRP could be further complicated by the variable length of follow-up that ranges from about three weeks to years in studies such as the Fenofibrate Intervention and Event Lowering in Diabetes (FIELD) study [16]. Studies with longer durations could have been influenced by extraneous unmeasured effects such as changes in body weight and other lifestyle attributes. Lastly, fenofibrate studies have used diverse participant pools with varied baseline serum CRP concentrations. Muhlestein et al. [13] have shown that individuals with high baseline CRP concentrations tend to experience a greater reduction in CRP than those with relatively low baseline values. Thus, it is possible that the benefit of fenofibrate is greater in people with elevated serum CRP concentrations in comparison to people with low serum CRP concentrations.

The aim of this study is to determine, through a meta-analysis, the effect of fenofibrate on serum CRP and to estimate the size of this effect in short-term trials (12 weeks or less) since they are unlikely to be confounded by long-term changes in body weight and behaviors such as diet and physical activity.

## 2. Methods

### 2.1 Literature searches

We conducted a comprehensive review of studies on the relation between fenofibrate and CRP that were published between 1966 and March 31, 2011. Using PubMed as the primary database, we searched for the following terms: "fenofibrate" and ("CRP" or "hsCRP" or "C-Reactive Protein" or "high-sensitivity C-Reactive Protein"). Search of additional databases including Google Scholar yielded studies that were already in PubMed. All full text articles that met the criteria below were independently retrieved and reviewed for study design and availability of relevant statistics by two reviewers (JY and JK). When duplicate studies were found, only the most complete article was included.

### 2.2 Selection criteria

Randomized clinical trials that met the following inclusion criteria were selected for the meta-analysis:

• Fenofibrate was one of the randomized treatment arms.

• Fenofibrate treatment lasted 3-12 weeks (short-term trial). Since most studies did not have a placebo and CRP can be affected by variables such as change in body weight, we restricted the analyses to short-term randomized studies.

• Findings were reported in the English language.

• Relevant statistics for imputing or computing the mean change and corresponding standard error were reported.

Any disagreements on the inclusion of identified studies by the two reviewers (JY and JK) were resolved by discussion or consensus involving a third reviewer (EK). The kappa statistic for the agreement between the first two reviewers was computed.

### 2.3 Data extraction

Two reviewers (JY and JK) independently extracted data from agreed upon studies using a standard data collection form. Characteristics of each study were recorded. These included the first author, country, publication year, dose and duration of fenofibrate treatment, sample size of fenofibrate treatment arm, patient population, comparison group, mean and standard error of the change in CRP concentration before and after fenofibrate treatment. Data sets extracted by the two reviewers were compared.

For some studies that reported median CRP concentrations before and after fenofibrate treatment, we imputed the mean change in CRP concentrations as the difference between median CRP concentrations before and after fenofibrate treatment. For some studies that did not report the standard error of the change in CRP concentration, we used the formulas below from the Cochrane's handbook for meta-analysis by Higgins *et al. *[19] to compute or impute the standard error:

• If the standard deviation (SD) was reported, we computed the standard error (SE) using $\text{SE}=\text{SD}∕\sqrt{N}$

• If the p-value was reported, we computed the t-statistic as t = Tinv (p-value, N-1), where Tinv(p-value, N-1) is a function that returns value t such that P(|X∥ > t) = p-value and X is a random variable that follows a t-distribution with N-1 degrees of freedom. The standard error was then obtained by dividing the mean change (MC) by the t value (SE = MC/t)

• If neither the SD of the mean change nor the p-value was reported but the standard errors of CRP concentration before and after treatment were reported, we imputed the SE of the change using a correlation coefficient from other studies using the formula below [19]:

$$corr=\frac{SD_{Before}^{2}+SD_{After}^{2}-SD_{Change}^{2}}{2\times SD_{Before}\times SD_{After}}$$

Using the sample size from each study as the weight, we computed a pooled correlation coefficient which is the weighted sum of correlation coefficients from all studies with enough information reported and then imputed the standard deviation of mean change as follows:

$$SD_{Change}=\sqrt{SD_{Before}^{2}+SD_{After}^{2}-2\times Corr\times SD_{Before}\times SD_{After}}$$

### 2.4 Meta-analysis

MIX software version 1.7 [20,21] was used for all analyses including generation of the Forest plot, Funnel plot and sensitivity analysis plot. To account for both within-study and between-study variation in the meta-analysis we used a random effects model with the inverse of the variance as the weight for each study. We tested for heterogeneity in the studies using Cochran's Q statistic and calculated I^2^, the percentage of the total variability in effect estimates among trials that is due to heterogeneity rather than chance. The summary effect of each study and the pooled effect from all studies are reported as point estimates and corresponding 95% confidence intervals on a Forest plot. To assess the potential for publication bias we used a Funnel plot. To evaluate whether an individual study had undue influence on the overall meta-analysis result, we performed sensitivity analyses by omitting one of the studies at a time and determining whether statistical conclusion remained the same. Studies were excluded one by one in the order in which they appear in the Forest plot. The study by Kim et al was excluded first and the study by Hogue et al was excluded last. To compute the number of null studies needed to invalidate the observed association from the meta-analysis (i.e., fail-safe *N*), we used formula number 3 from Orwin [22]. We computed fail-safe *Ns *for the small and medium Cohen's criterion values (*d_c_*) of 0.2 and 0.5, respectively.

## 3. Results

### 3.1. Literature searches

**Figure 1:**
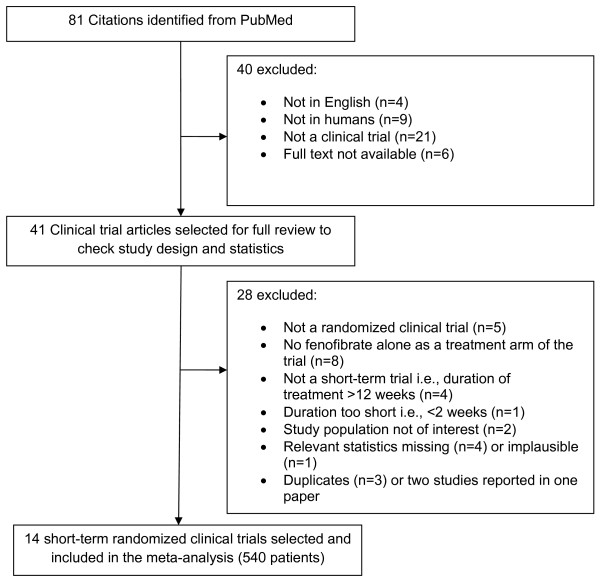
**Flow chart showing how studies were selected for inclusion in the meta-analysis**.

We identified a total of 81 citations in PubMed. As shown in Figure 1, 77 of the studies were published in English, from which we excluded 9 that were not done in humans, 21 that were review articles or not clinical trials and 6 whose full texts we could not obtain. Finally, 41 articles were fully reviewed for study design and relevant statistics. Based on our criteria for study selection, 5 of these were not randomized clinical trials, 4 were long-term studies (> 12 weeks of fenofibrate treatment), 2 had populations that were not of interest given potential confounding by medications (e.g., steroids in rheumatoid arthritis), 4 did not report sufficient statistics for our meta-analysis, one study was too short (lasted < 2 weeks), one reported statistics that seemed implausible and 3 were duplicates. After all the exclusions, 14 studies with a total of 540 patients met our criteria for inclusion in the meta-analysis as summarized in Figure 1 and Tables 1 and 2.

**Table 1:** Summary of the 14 randomized clinical trials included in the meta-analysis on the short-term effect of fenofibrate on CRP.

Index	Country	First author and publication year	Dose (mg/day)	Duration (weeks)	Sample size	Population	Randomized?
1	Korea	Kim *et al.*, 2006 [40]	200	8	54	Patients with a triglyceride level > 200 mg/dL	Yes (Control group is general measures with low-calorie and low-fat diet and aerobic exercise)
2	UK	Fegan *et al.*, 2005 [17]	200	12	10	Ambulatory male or female patients aged 40-75 years with Type 2 diabetes for more than 2 years	Yes (Compared with placebo)
3	Poland	Undas *et al. *2005 [41]	160	8	22	Patients with LDL > 130 mg/dl and triglycerides < 300 mg/dl	Yes (Compared with Simvastatin)
4	Slovenia	Sebestjen *et al.*, 2004 [42]	250	12	38	Healthy male volunteers, non-smokers, aged between 40 and 60 years, with combined hyperlipidemia	Yes (Compared with cerivastatin)
5	Taiwan	Wang *et al.*,2003 [43]	200	8	35	Triglyceride between 200 and 500 mg/dl, cholesterol > 200 mg/dl, and cholesterol/HDL ratio > 5	Yes (Compared with Simvastatin)
6	Korea	Wi *et al.*, 2010 [15]	160	8	80	Patients with triglyceride levels of 150-499 mg/dL, HDL levels < 45 mg/dL and LDL levels < 130 mg/dL	Yes (Compared with niacin)
7	Porland	Krysiak *et al.*, 2010 [44]	200	12	61	Metabolic syndrome with pre-diabetes	Yes (Compared with placebo)
8	Porland	Krysiak *et al.*, 2010 [44]	200	12	19	Metabolic syndrome without pre-diabetes	Yes (Compared with placebo)
9	USA-MI	Rosenson *et al.*, 2010 [45]	160	12	25	Patients with the metabolic syndrome	Yes (Compared with placebo)
10	USA-TX	Belfort *et al. *2010 [46]	200	12	16	Nondiabetic insulin-resistant MetS subjects	Yes (Compared with placebo)
11	Poland	Pruski *et al.*, 2009 [24]	267	8	30	Type 2 diabetic patients with mixed dyslipidemia	Yes (Compared with placebo)
12	USA-UT	Muhlestein et al., 2006 [13]	160	12	100	Patients with type 2 diabetes, mixed dyslipidemia and no history of coronary heart disease	Yes (Compared with Simvastatin)
13	Poland	Okopien *et al.*, 2006 [32]	267	4	31	Patients with impaired glucose tolerance	Yes (Compared with placebo)
14	Canada	Hogue *et al.*, 2008 [18]	200	6	19	Type 2 diabetes mellitus subjects with hypertriglyceridemia	Yes (Compared with atorvastatin)

**Table 2:** Summary of the reported CRP change and the sponsors for the studies included in the meta-analysis on the short-term effect of fenofibrate on CRP

Index	Country	Study Name	Baseline CRP (mg/L)†	Post-treatment CRP (mg/L)†	Sponsor
1	Korea	Kim *et al.*, 2006 [40]	1.74 ± 1.74	1.54 ± 1.66	No mention
2	UK	Fegan *et al.*, 2005 [17]	3.27 ± 1.71	4.03 ± 3.13	Bayer pharmaceuticals
3	Poland	Undas *et al. *2005 [41]	2.33 ± 2.81	1.45 ± 1.41	Polish committee for scientific research and Merck Sharp & Dohme & Fournier Laboratories
4	Slovenia	Sebestjen *et al.*, 2004 [42]	1.60 (1.10, 3.30)*	1.10 (0.70, 2.70)*	No mention
5	Taiwan	Wang *et al.*,2003 [43]	1.70 (0.90, 2.30)*	1.30 (0.70, 2.00)*	National Taiwan University Hospital, National Science Council and the Academia Sinica (China)
6	Korea	Wi *et al.*, 2010 [15]	1.20 (0.43, 3.86)*	0.90 (0.30, 4.86)*	Ministry of Health and welfare (Korea), R&BD program (Korea) and Cardiovascular Research Center (Korea)
7	Poland	Krysiak *et al.*, 2010 [44]	2.4 ± 1.56	1.20 ± 0.78	Committee of Scientific Research
8	Poland	Krysiak *et al.*, 2010 [44]	1.70 ± 0.87	0.90 ± 0.44	Committee of Scientific Research
9	USA-MI	Rosenson *et al.*, 2010 [45]	2.60 (1.40, 3.10)*	1.90 (1.10, 2.60)*	Abbott Laboratories
10	USA-TX	Belfort *et al. *2010 [46]	6.50 ± 6.00	3.60 ± 3.60	Veterans Admission Research Career development award, Howard Hughes Medical Institute, the Veterans Affairs Medical Research Fund and the National Center for Research Resources
11	Poland	Pruski *et al.*, 2009 [24]	2.8 ± 1.10	1.90 ± 2.19	Medical University of Silesia
12	USA-UT	Muhlestein et al., 2006 [13]	1.99**	1.45**	No mention
13	Poland	Okopien *et al.*, 2006 [32]	2.6 ± 1.27	1.95 ± 1.27	Medical University of Silesia
14	Canada	Hogue *et al.*, 2008 [18]	2.97 ± 3.29	2.86 ± 3.58	Pfizer

†Figures reported as mean ± standard deviation; * Figures reported as median (25^th ^quartile, 75^th ^quartile); **Only medians reported.

### 3.2. Efficacy

**Figure 2:**
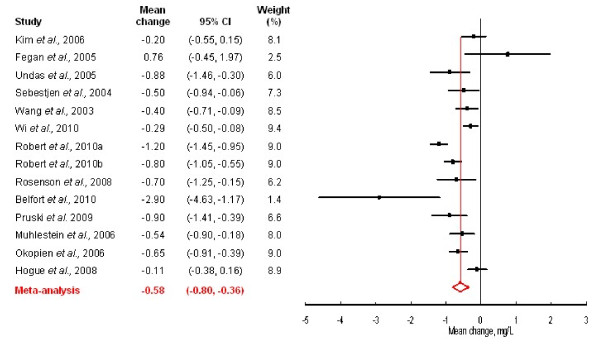
**Forrest plot showing the change in C-reactive protein concentrations following treatment with fenofibrate**.

The weighted mean CRP concentrations before and after fenofibrate therapy were 2.15 mg/L and 1.53 mg/L (-28.8% change), respectively. As shown in Figure 2, short-term treatment with fenofibrate significantly lowers CRP by 0.58 mg/L (95% CI: 0.36-0.84 mg/L). There was significant heterogeneity (Q statistic = 64.5, *P *< 0.0001) in the studies. The high estimated I^2 ^value (79.8%) confirmed the need to use a random-effects meta-analysis model as we did in this study.

Studies included in the current analyses were conducted in various parts of the world and included participants that varied in their baseline CRP concentrations. We thus reanalyzed the data after stratifying studies by region or baseline CRP concentrations. Seven of the studies were from Europe, 4 from North America and 3 from Asia. The resultant pooled estimates (95% CI) for CRP reduction were -0.72 (-1.01, -0.42; *P *< 0.0001) for Europe, -0.61 (-1.14, -0.08; *P *= 0.02) for North America and -0.30 (-0.46, -0.14; *P *= 0.0002) for Asia. In agreement with previous studies [13], we considered a baseline CRP ≥ 2.0 mg/L to be high. We then carried out an analysis restricted to the studies that had participants with median/mean baseline CRP ≥ 2.0 mg/L [13]. As expected, we observed a larger decrease in CRP following treatment in individuals with high baseline CRP. The pooled estimate from 8 studies with high CRP was -0.72 (95% CI: -1.12, -0.32) mg/L compared to 0.58 (95% CI: 0.36-0.80) mg/L in analyses that pooled all the 14 studies.

### 3.3. Publication Bias

**Figure 3:**
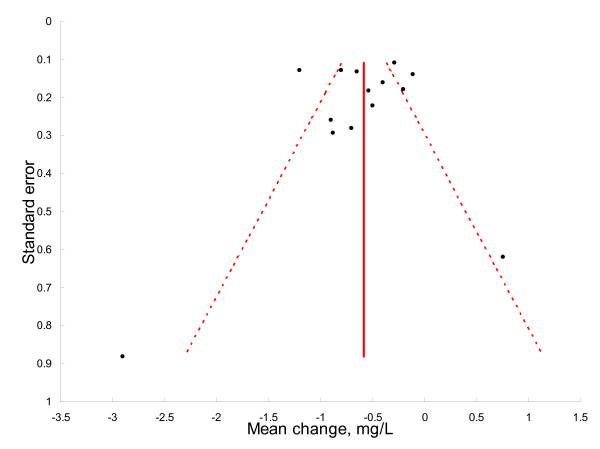
**Funnel plot showing no evidence for publication bias**.

The Funnel plot (Figure 3) is fairly symmetric, suggesting no evidence for publication bias.

### 3.4. Sensitivity analysis

**Figure 4:**
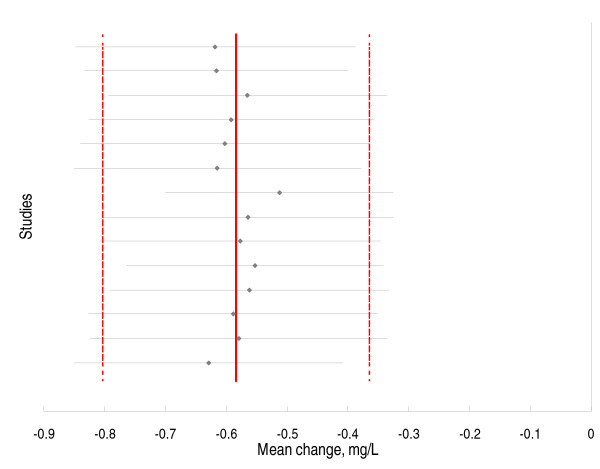
**Effect of excluding one study at time on the overall estimated effect of fenofibrate on C-reactive protein concentrations**. The solid line indicates the pooled estimate while the dashed lines indicate the 95% confidence intervals. The studies were excluded from the analysis in the order they appear in the Forest plot (Figure 2) i.e., the study by Kim et al was excluded first and the study by Hogue et al was excluded last.

Sensitivity analysis (Figure 4) suggests that by excluding any of the 14 studies from the meta-analysis the estimated effect will still be within the 95% CI of the estimated effect under the null hypothesis and will not get a different conclusion from the overall meta-analysis result.

### 3.5. Kappa and fail-safe N

The Kappa for agreement in selecting studies for inclusion in the meta-analysis was 0.47. The fail-safe *Ns *for the small and medium Cohen's criterion values were 64 and 17 studies, respectively.

## 4. Discussion

The results from the current meta-analysis show that short-term treatment with fenofibrate leads to a significant reduction in serum CRP concentrations (0.58 mg/L or 28.8% reduction, *P *< 0.0001) and that the magnitude of the reduction is significantly heterogeneous across studies (Q = 64.5; *P *< 0.0001). Using the random effects model, the pooled mean absolute reduction of serum CRP was 0.58 mg/L (95% CI: 0.36-0.80) with a Z-score for the overall effect of 12.3 (*P *< 0.0001). The mean reduction in CRP varied by region and was greatest for studies that had higher baseline CRP concentrations. Sensitivity analyses show that the overall effect is robust since none of the studies has an undue effect on our study findings and stratification by region did not change the conclusion from this study. Our findings do not seem to be affected by publication bias as shown by the Funnel plot.

To date, there is no published meta-analysis on the relation between fenofibrate and CRP. In agreement with our meta-analytic findings, many short-term studies have reported a significant reduction in serum CRP following fenofibrate therapy [23-26]. In support of our finding that fenofibrate reduces CRP concentrations in serum, there are mechanistic human studies showing that fenofibrate therapy reduces the production of inflammatory cytokines by monocytes [14]. Other short-term studies have reported a non-significant reduction in serum CRP concentration following administration of fenofibrate [27]. Some long-term studies have also reported significant reductions in serum CRP following fenofibrate therapy [28] while others reported a non-significant reduction in serum CRP concentrations [29]. However, not all studies investigating the relationship between fenofibrate and serum CRP have reported findings that agree with our findings. Some short-term [17] and long-term [16] studies such the FIELD study have reported an increase in serum CRP following fenofibrate therapy.

The reasons for the disparity in the response to fenofibrate, especially in short-term studies where correlates of CRP such as BMI remain stable, remain obscure. One possible reason for this apparent variability in study findings is that many of the studies have had the disadvantage of having small sample sizes (usually < 50 patients) thus making their estimates of effect likely to be unstable, especially if the baseline CRP concentrations are very low. This disadvantage of small sample sizes could have been overcome by the fairly large sample size of 540 patients in 14 studies used in the current meta-analysis. Moreover, the estimated effect of fenofibrate on CRP is a weighted average, which gives a more precise estimate than any of the individual studies upon which the meta-analysis is based. Also, participants in studies on fenofibrate and CRP were recruited based on their triglyceride concentrations. Although most patients with high triglycerides tend to have high CRP, this is not always the case and may confound results given that lipid-lowering drugs tend to lower CRP more among individuals who have baseline CRP concentrations above 2 mg/L [13] and may increase CRP in patients with very low baseline concentrations. Indeed our analyses restricted to studies with CRP concentrations ≥ 2 mg/L showed that the reduction in CRP in this subset (-0.72; 95% CI: -1.12, -0.32 mg/L) was greater than that observed when all studies are combined (0.58; 95% CI: 0.36-0.80 mg/L). We also observed regional differences in CRP reduction. The reduction in CRP observed for studies conducted in Europe was similar to that observed for studies in North America but much greater than that observed for studies conducted in Asia. The reason is not clear but may in part reflect the differences in baseline CRP and small number of studies conducted in Asia (n = 3) compared to those conducted in Europe (n = 7) or North America (n = 4). However, despite variability by region, all the three pooled estimates indicate a significant reduction in CRP following treatment with fenofibrate. Evaluation of the efficacy of fenofibrate on serum CRP will require randomized studies that recruit patients with high baseline CRP concentrations e.g., ≥ 2 mg/L.

The other potential reason for the variability in findings across studies could be the variation in the strength of fenofibrate used. The dosage of fenofibrate used in most short-term studies we reviewed varied from 145 mg/day [30,31] to 267 mg/day [32,33]. Furthermore, various formulations (e.g., micronized vs. non-micronized) were used. Although, different formulations are expected to be metabolically equivalent, this may not always be the case given potential variability in adherence and genetic effects on response to fenofibrate. Since fenofibrate acts mainly through binding onto peroxisome proliferator-activated receptor-alpha [34], it is possible that formulations that result in lower amounts of the available active form of fenofibrate will lead to different physiological outcomes and this could explain the observed inconsistencies across studies. In addition to the differences in dosage and/or formulations, most fenofibrate studies on CRP either did not include a placebo arm or used another lipid-lowering drug as the comparison group. Since randomized placebo-controlled studies on fenofibrate are very few, it is difficult to disentangle the true effects of fenofibrate from those that are simply due to regression to the mean, especially given the small size of most short-term fenofibrate trials.

Another potential reason for the variability in the outcome could be the study duration. There is evidence that the effect of fenofibrate on clinical parameters may not be complete within 3 weeks of treatment with fenofibrate (Kabagambe et al., unpublished findings) and this period could be longer as shown in other studies [14]. Other unmeasured variables that influence serum CRP concentrations could change during long-term follow-up but most studies did not have a placebo arm to investigate this effect. However, this was not the case in the Helsinki arm of the Fenofibrate Intervention and Event Lowering Diabetes (FIELD) trial which included a placebo and where individuals were randomized [16]. In this study that included 170 patients with relatively low CRP concentrations (median ≤ 1.8 mg/L), fenofibrate therapy was associated with a 20.1% non-significant increase in CRP (95% CI: -0.8 to 28.8%) over a 5-year follow-up period [16]. Since most studies did not have a placebo group we restricted our analyses to short-term studies (≤ 12 weeks) to minimize potential changes in CRP that are due to changes in patient characteristics e.g., BMI.

Our findings present significant clinical implications in the management of elevated serum concentrations of CRP. To date, only one clinical trial (JUPITER) where patients were recruited based on having high CRP concentrations (≥ 2 mg/L) has been reported [35]. This study [35] showed that compared to the placebo, rosuvastatin lowered CRP by 37%, a magnitude higher than that observed in the current meta-analysis (28.8% mean reduction) of studies with a wide range of baseline CRP concentrations. Whether the effect of fenofibrate among patients with high CRP is comparable to that of resuvastatin is yet to be established. Studies comparing other statins to fenofibrate, showed that among patients with CRP > 2 mg/L, the reduction in CRP due to fenofibrate (-19.0%) was comparable to that due to simvastatin (-24.8%) therapy [13]. In another study [14] the reduction in CRP due to fenofibrate therapy (23 ± 4%) was comparable to that by simvastatin (20 ± 3%), but the patient populations in the two groups were somewhat different with regard to their lipid profiles at study entry. Most studies comparing fenofibrate to other lipid-lowering medications were quite small in sample size (< 50 patients). Until equivalence studies with rosuvastatin as a reference are done, it is plausible to have rosuvastatin as the established approach for managing elevated CRP concentrations. However, some patients experience adverse reactions following administration of statins [36]. Fish oil or fish oil esters are the other option but have side effects too such as undesirable fish odor or increased risk for bleeding and in some studies, including randomized trials, reduction in CRP was not attained [37]. Niacin is the other option and in one study [15] the magnitude of reduction in CRP was higher than that of fenofibrate but as for some statins [38], use of niacin has been associated with glucose dysregulation [15].

Our results confirm that fenofibrate is another feasible alternative therapy in the short-term management of CRP especially among patients who do not tolerate statins, fish oil products, niacin or are unable to adhere to increased physical activity or healthy lifestyle changes. Whether reduction of CRP will be observed among patients with high baseline CRP and followed over a long period as in the ACCORD (Action to Control Cardiovascular Risk in Diabetes) study [39] is yet to be determined.

We acknowledge a number of limitations in our study. First, we used secondary data and thus are unable to rule out potential errors in the original studies. Such errors, if existent, they could bias our conclusions but the sensitivity analyses showed that the conclusion from this meta-analysis is not sensitive to elimination of an individual study. Secondly, although we have attempted to explore publication bias as a potential explanation of the findings, we did not find evidence for it. We believe that publication bias is unlikely to have affected our findings because standard inclusion and exclusion criteria were used in all the studies thus making preferential exclusion/inclusion highly unlikely. Moreover, the computed fail-safe *N *suggests that we would need 17-64 null studies to make the current meta-analysis show a null association between fenofibrate and CRP.

## 5.0 Conclusion

We conclude that short-term treatment with fenofibrate is an effective strategy in the management of elevated CRP concentrations. Despite having significant heterogeneity in the studies analyzed, this meta-analysis has shown a significant pooled mean reduction in serum CRP of 0.58 mg/L (95% CI: 0.36-0.80; *P *< 0.0001). Our findings have significant clinical implications in that fenofibrate is an option for patients who cannot tolerate statins, niacin or fish oil products and their derivatives. Although, fenofibrate has not shown benefit on all-cause mortality, it may be desirable to conduct a trial among people with elevated CRP but who do not tolerate therapies such as statins.

## List of abbreviations

CRP: C-reactive protein; SD: Standard deviation; SE: Standard error.

## Competing interests

The authors declare that they have no competing interests.

## Authors' contributions

EK conceived the idea. All authors refined the idea and JY and JK searched and extracted data from PubMED. JY, JK and EK performed the analyses. JY and JK drafted the manuscript. All authors edited various copies of the manuscript and approved the final version.
